# Immunohistochemical Analysis of Potential Therapeutic Targets PRAME, FOLR1, and CLDN18.2 in Salivary Gland Carcinomas

**DOI:** 10.1002/cam4.71799

**Published:** 2026-04-12

**Authors:** Lukas A. Brust, Jan Philipp Kühn, Sandrina Körner, Moritz Knebel, Felix L. Braun, Philippe Zeidan, Silke Wemmert, Bernhard Schick, Sigrun Smola, Mathias Wagner, Martin Ertz, Maximilian Linxweiler

**Affiliations:** ^1^ Department of Otorhinolaryngology, Head and Neck Surgery Saarland University Homburg Germany; ^2^ Institute of Virology Saarland University Homburg Germany; ^3^ Department of General and Surgical Pathology Saarland University Homburg/Saar Germany

**Keywords:** CLDN18.2, FOLR, immunohistochemistry, PRAME, salivary gland carcinoma

## Abstract

Salivary gland carcinomas are rare, heterogeneous, and often resistant to conventional treatments, highlighting the need for new therapeutic strategies. This retrospective study evaluated the expression of three immunologically relevant biomarkers—PRAME, FOLR1, and CLDN18.2—as potential therapeutic targets in salivary gland carcinomas. Tumor samples from 54 patients with seven histological subtypes treated at Saarland University Medical Center between 2013 and 2023 were analyzed using immunohistochemistry and scored with the Immunoreactive Score. PRAME was strongly expressed in 89% of cases, while FOLR1 and CLDN18.2 showed markedly lower expression at 41% and 6%, respectively. High PRAME expression was significantly associated with the presence of distant metastases, regardless of histological subtype. Patients with low PRAME expression tended to have improved overall survival, as indicated by Kaplan–Meier analysis and Log‐Rank testing. These findings suggest PRAME as a promising immunotherapeutic and diagnostic target for salivary gland carcinomas, particularly through T‐cell‐based therapies already under investigation in other malignancies such as acute myeloid leukemia. Further preclinical studies are needed to validate the functional and therapeutic significance of PRAME, FOLR1, and CLDN18.2 in this context.

## 1 Introduction

Salivary gland carcinomas (SGC) are a rare and highly heterogeneous group of human malignancies, with more than 20 histological subtypes [1]. SGCs comprise 20% of parotid, 50% of submandibular, and 80% of minor salivary gland tumors [2]. Common histological subtypes of SGCs, as classified by the WHO Classification of Head and Neck Tumors (5th edition), include salivary duct carcinoma, adenoid cystic carcinoma, mucoepidermoid carcinoma, epithelial‐myoepithelial carcinoma, acinic cell carcinoma, and polymorphous adenocarcinoma [3]. These subtypes are partially characterized by distinct molecular and genetic alterations [4].

SGCs pose a significant clinical challenge due to their rarity, heterogeneous histological characteristics, and aggressive clinical behavior [5]. Standard treatment modalities—surgery, radiotherapy, and chemotherapy—frequently yield limited success, emphasizing the need for novel, targeted therapeutic strategies [6, 7, 8]. Recurrent or metastatic SGCs remain challenging to treat, with limited curative options and no disease‐specific FDA‐approved therapies [9, 10]. However, treatments, such as Trastuzumab deruxtecan for HER2‐overexpressing tumors or pembrolizumab for tumors with a high tumor mutational burden (TMB > 10 mutations/megabase) [11], offer potential therapeutic approaches for selected patients [12, 13]. Despite these advances, outcomes are often poor, and therapeutic options remain largely palliative.

In recent years, the identification of immunological targets has gained considerable attention. PRAME (Preferentially Expressed Antigen in Melanoma) has already been investigated as a potential target for immunotherapy in various tumor types, including melanoma, endometrial carcinomas, uterine serous carcinomas, ovarian carcinomas, and seminomas [14]. Emerging immunotherapeutic approaches, such as T‐cell‐based therapies and PRAME‐targeting vaccines, are being explored for their cancer‐specific potential [15]. PRAME is a nuclear receptor and transcriptional regulator belonging to the cancer‐testis antigen family [16]. Under physiological conditions, PRAME is restricted to testicular tissue, where it represses the retinoic acid receptor pathway, influencing cell differentiation, growth, and apoptosis [17, 18]. However, it exhibits high expression in various cancer entities [19, 20, 21].

Alongside PRAME, this study evaluated FOLR1 (Folate Receptor 1) and CLDN18.2 (Claudin 18.2) to determine their expression patterns and prognostic significance in SGCs. Folate receptors, including FOLR1 and FOLR2, are key folate transporters that are overexpressed in various cancers [22]. Furthermore, FOLR1 is linked to tumor progression and poor prognosis [22].

CLDN18.2 is a tight junction protein exclusively expressed in normal gastric mucosa cells and is retained in most gastric and gastroesophageal junction (G/GEJ) adenocarcinomas. In normal gastric mucosa, CLDN18.2 is typically confined within tight junctions. However, during malignant transformation, the loss of cell polarity exposes CLDN18.2, making it accessible to therapeutic antibodies and a promising target for upcoming immunotherapies [23].

Recent advances in targeted therapy have highlighted PRAME, FOLR1, and CLDN18.2 as promising candidates due to their overexpression in malignancies and restricted presence in normal tissue. PRAME has emerged as a pan‐cancer target, supported by systematic reviews [14] and ongoing clinical trials investigating autologous T cell therapies in PRAME‐positive advanced solid tumors [24]. FOLR1 has likewise gained clinical relevance, as underscored by the FDA approval of mirvetuximab soravtansine‐gynx, an antibody‐drug conjugate for FRα‐positive, platinum‐resistant ovarian cancer [25]. CLDN18.2, normally restricted to the gastric mucosa, is exposed in malignant transformation and is currently under investigation in phase III trials with the monoclonal antibody zolbetuximab in gastric and gastroesophageal cancers [26]. Although mechanistically distinct, the unifying rationale behind the selection of these targets is their tumor‐specific expression and their potential to be targeted for future immunotherapeutic approaches. Given the lack of established biomarkers in SGCs, their evaluation in this tumor entity is both novel and clinically relevant.

The primary objective of this study was to determine the expression profiles of these three therapeutically relevant biomarkers in a cohort of 54 SGC patients and to analyze their correlation with clinical and histopathological patient characteristics, including overall survival.

## 2 Materials & Methods

### 2.1 Patient Cohort and Study Design

This retrospective study included 54 patients with histologically confirmed malignant salivary gland tumors treated at Saarland University Medical Center (Homburg/Saar, Germany) between 2013 and 2023. Demographic data, tumor histology, and clinical follow‐up information were obtained from institutional records. All patients provided written informed consent for the scientific use of their tissue samples and clinical data. The study was approved by the Saarland Ethics Review Board (index number 280–10) and conducted in accordance with the Declaration of Helsinki and relevant guidelines. The cohort comprised 30 male and 24 female patients. Tumor staging was classified according to the 8th edition of the AJCC TNM system. Additional epidemiological and clinical characteristics are summarized in Table 1. This study analyzed treatment modalities, including surgery, radiotherapy (RT), and radiochemotherapy (R(C)T). Tumor tissue samples were collected either during diagnostic biopsy for histological verification or therapeutic tumor resection.

**TABLE 1:** Demographic and clinical patient data.

		SGC patients
No. of patients		54
Sex	Male	30 (55.6%)
	Female	24 (44.4%)
Localization	Parotid Gland	42 (77.7%)
	Sumandibular Gland	10 (18.6%)
	Minor Salivary Glands	2 (3.7%)
Histological Subtype	Adenoid Cystic Carcinoma (ACC)	15 (27.8%)
	Acinic Cell Carcinoma (AZC)	11 (20.4%)
	Adenocarcinoma, NOS not otherwise specified (ADC)	7 (12.9%)
	Mucoepidermoid Carcinoma (MEC)	7 (12.9%)
	Salivary Duct Carcinoma (SDC)	7 (12.9%)
	Myoepithelial Carcinoma (MC)	5 (9.3%)
	Oncocytic Carcinoma (OZC)	2 (3.8%)
T* stage	1	17 (31.5%)
	2	19 (35.2%)
	3	12 (22.2%)
	4	6 (11.1%)
N* stage	0	35 (64.8%)
	1	5 (9.3%)
	2	10 (18.5%)
	3	4 (7.4%)
M* stage	0	41 (75.9%)
	1	13 (24.1%)
AJCC* Stage	I	14 (25.9%)
	II	13 (25.1%)
	III	5 (9.2%)
	IV	22 (40.8%)

*Note:* ^*^NThe 8th version of the TNM/AJCC classification was used to categorize the carcinomas. Histopathological diagnoses were established according to the 4th edition of the WHO classification.

### 2.2 Immunohistochemical Analysis

Formalin‐fixed, paraffin‐embedded (FFPE) tissue samples from included patients were used for histopathological and immunohistochemical analyses of PRAME, FOLR1, and CLDN18.2 expression in SGCs. Fresh tissue samples were first fixed in PBS‐buffered 4% formalin for 24 h before embedding in paraffin using Tissue‐Tek VIPTM5 JR (Sakura Finetek, Ulmkirch, Germany). FFPE tissue sections were then prepared for immunohistochemical staining. Initially, three 10 μm sections were discarded before obtaining 3 μm‐thick sections using a Leica RM2235 rotary microtome (Leica Microsystems, Wetzlar, Germany). Sections were transferred onto Superfrost Ultra Plus microscope slides (Menzel‐Gläser, Braunschweig, Germany) and dried overnight at 37°C.

Immunohistochemistry (IHC) was conducted using the automated VENTANA BenchMark Ultra instrument (Roche, Basel, Switzerland). The following assays and antibodies were employed: VENTANA FOLR1 (FOLR1‐2.1) RxDx assay, VENTANA CLDN18 (43‐14A) assay, and the anti‐PRAME (EPR20330) rabbit monoclonal primary antibody. Visualization was performed using the OptiView DAB IHC Detection Kit according to the manufacturer's protocol. Additionally, for the double staining, we used the polyclonal Rabbit Anti‐S‐100 primary antibody (Agilent, Hamburg, Germany; Cat. No. Z0311) at a dilution of 1:10,000, in combination with the UltraView Universal Alkaline Phosphatase Red Detection Kit (Ventana Medical Systems, Tucson, AZ, USA). All staining procedures were performed according to the manufacturers' protocols. The double staining was performed on a representative SGC specimen to visualize perineural invasion of the carcinomas.

**FIGURE 1:**
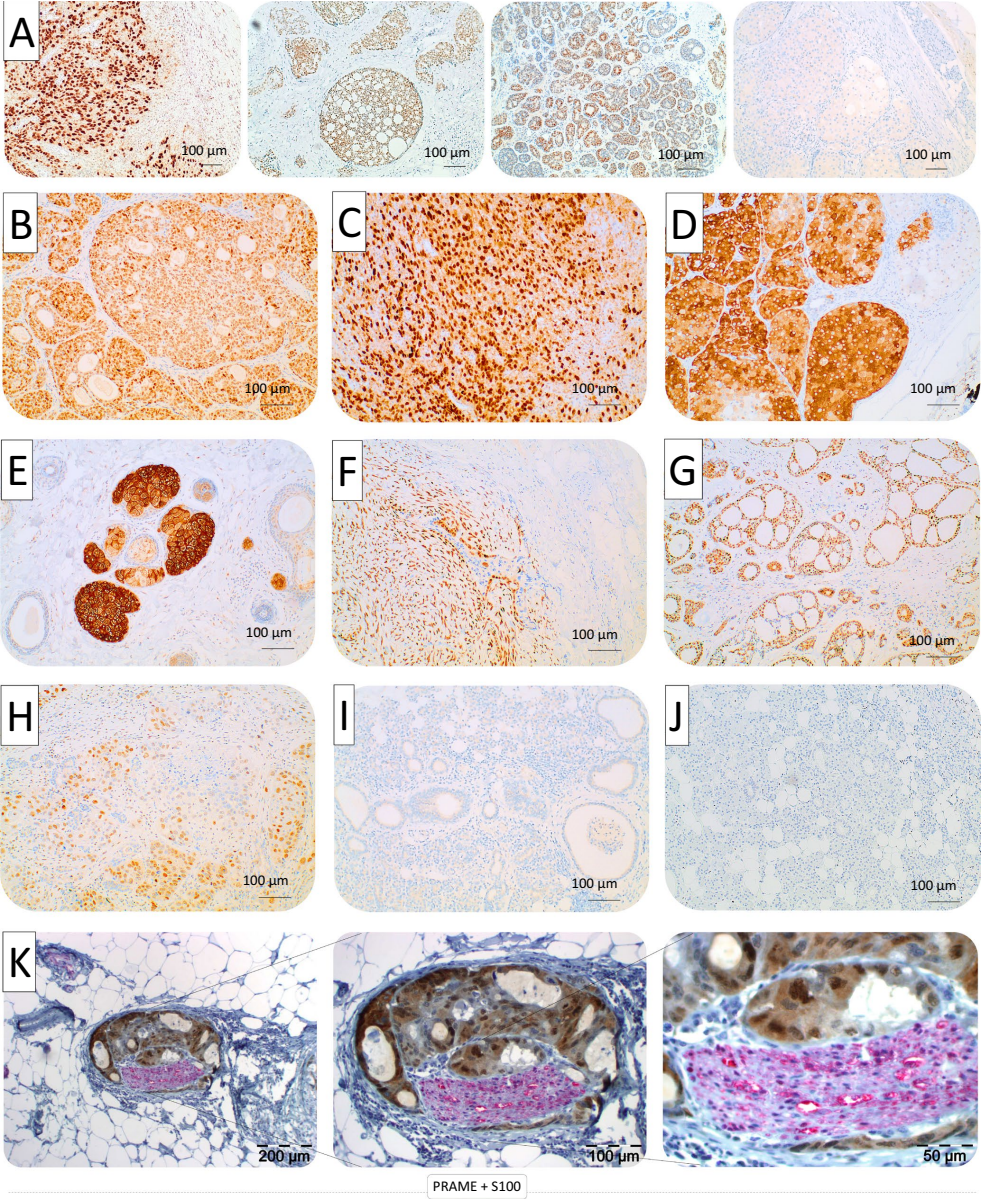
(A) Representative images of PRAME immunostaining in SGCs, demonstrating varying patterns of expression. From left to right: (i) strong staining intensity with diffuse positivity in nearly all tumor cells, (ii) moderate intensity with widespread positivity, (iii) strong intensity limited to approximately 50% of tumor cells, and (iv) negative case with no detectable immunoreactivity. Positive immunostaining is indicated by brown coloration. (B–H) Immunohistochemical staining of PRAME‐positive SGCs: (B) Myoepithelial Carcinoma (MC), (C) Acinic cell carcinoma (AZC), (D) Oncocytic carcinoma (OZC), (E) Mucoepidermoid carcinoma (MEC), (F) Adenocarcinoma, NOS (ADC), (G) Adenoid cystic carcinoma (ACC), (H) Salivary duct carcinoma (SDC). (I) Immunohistochemical staining of PRAME‐negative SDC. (J) Physiological, tumor‐free salivary gland tissue, serving as a negative control. No immunostaining was observed for PRAME, FOLR1, or CLDN18.2, confirming a specific expression in SGC tissue. (K) Double staining with PRAME (brown) and S100 (red) of an ACC case with perineural invasion of the facial nerve at different magnifications, highlighting tumor‐specific PRAME expression.

A semiquantitative immunohistochemical analysis was conducted using the Immunoreactivity Score (IRS) as described by Remmele and Stegner (1987) [27]. IRS values (0–12) were assigned based on staining intensity and the percentage of positive tumor cells: Staining intensity: None (0), weak (1), moderate (2), strong (3). Percentage of positive cells: 0% (0), < 10% (1), 10%–50% (2), 51%–80% (3), > 80% (4). The final IRS was obtained by multiplying both values, resulting in a range from 0 (negative) to 12 (strongly positive). Immunoreactivity of PRAME, FOLR1, and CLDN18.2 was assessed exclusively in tumor cells. Tumor regions were defined based on histopathological landmarks, with intratumoral areas comprising tumor cell nests, as illustrated in Figure 1. To compare the expression of PRAME, FOLR1, and CLDN18.2 in SGC with physiological salivary gland tissue, immunohistochemical staining was performed on tumor‐free salivary gland tissue, which was found in 51 out of 54 samples. Accordingly, FFPE sections of non‐neoplastic salivary gland tissue adjacent to the malignant samples were analyzed using the same staining protocol. Marker expression was assessed exclusively in epithelial cells, with subcellular localization recorded according to the biological characteristics of each marker. PRAME expression was evaluated as nuclear staining, whereas FOLR1 and CLDN18.2 were evaluated with emphasis on intracellular and membranous staining.

Immunohistochemical stainings were independently analyzed by three investigators, including a board‐certified pathologist. Histopathological diagnoses were established according to the 4th edition of the WHO classification valid at the time of diagnosis. For statistical analysis, the arithmetic mean of the three IRS values per sample was used. High or low expression levels were determined using the mean IRS value of all samples as a diagnostic threshold.

### 2.3 Statistical Analysis

For statistical analysis, Prism 9 software (GraphPad Software, Boston, MA, USA) was used. Normal distribution of the data was assessed using the Anderson–Darling, D'Agostino & Pearson, Shapiro–Wilk, and Kolmogorov–Smirnov tests. If at least two tests indicated a normal distribution, parametric tests were applied (unpaired *t*‐test with Welch's correction, one‐way ANOVA). If these criteria were not met, non‐parametric tests were used (Mann–Whitney *U* test, Kruskal–Wallis test). Overall survival (OS) analysis was performed using the Mantel–Cox test (log‐rank test) and visualized with Kaplan–Meier curves. A *p*‐value < 0.05 was considered statistically significant (α = 0.05). The specific statistical tests used are indicated in the respective figure legends or the main text.

## 3 Results

### 3.1 PRAME Expression in SGC and Histological Characteristics

Immunohistochemical analysis revealed distinct patterns of PRAME expression in SGCs, as illustrated in Figure 1. Positive PRAME staining was exclusively identified within intratumoral regions while normal salivary gland tissue consistently showed no expression of PRAME, FOLR1, or CLDN18.2 (Figure 1J). This tumor‐specific reaction confirmed the tumor‐associated nature of PRAME expression.

**FIGURE 2:**
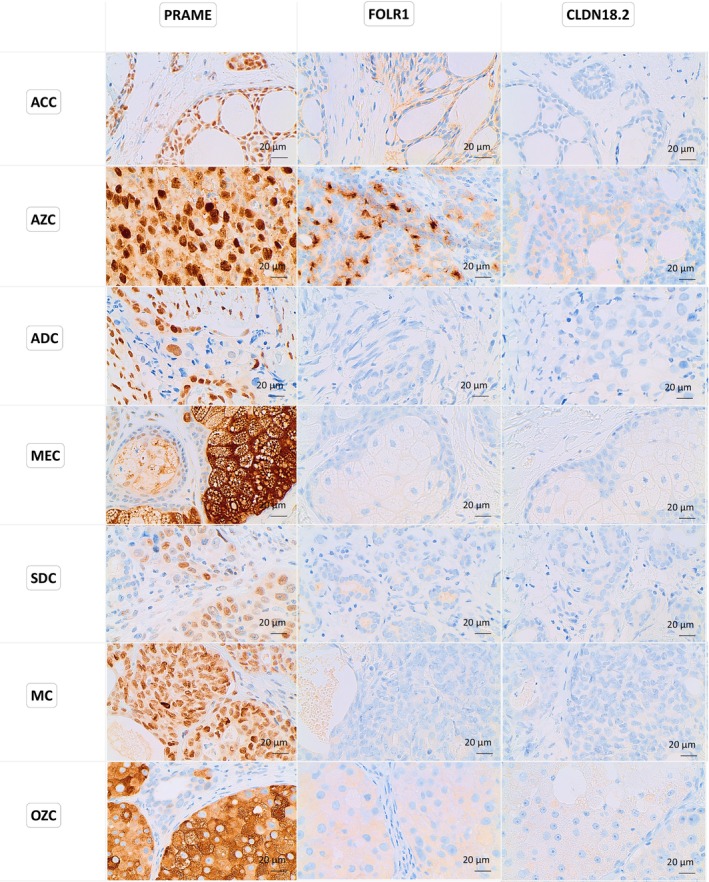
Representative immunohistochemical staining of PRAME, FOLR1, and CLDN18.2 in SGCs at 40× magnification. PRAME exhibits predominantly nuclear staining, while FOLR1 and CLDN18.2 show cytoplasmic localization.

Figure 1A shows representative images of PRAME immunostaining in SGC, showcasing varying staining intensities. These ranged from strong intensity, with nearly all tumor cells being positive, to no staining reaction in negative specimens. Positive immunostaining appeared brown, highlighting the differential expression of PRAME across tumor samples. Figure 2 further illustrates the immunohistochemical staining patterns of PRAME, FOLR1, and CLDN18.2 in SGC, emphasizing the heterogeneity of expression among tumor samples.

Adenoid cystic carcinoma (ACC), a subtype of SGC, was particularly notable for its histological characteristics, including perineural invasion, as shown in Figure 1K. Double staining with PRAME (brown) and S100 (red) in ACC with perineural invasion of the facial nerve highlighted PRAME expression at different magnifications. The high immunoreactivity score in these regions, with a maximum possible score of 12, underscores the robust expression of PRAME in ACC. This subtype, along with others such as AZC, ADC, MEC, OZC, and ADC, exhibited distinct histological features that showed PRAME expression. For instance, ACC was characterized by its cribriform and tubular patterns, while MEC often displays a combination of mucous, epidermoid, and intermediate cells. All histological subtypes demonstrated positive immunoreactivity for PRAME (Figure 1B–H).

### 3.2 PRAME Is Highly Expressed in SGC and Associated With Progressive Disease

**FIGURE 3:**
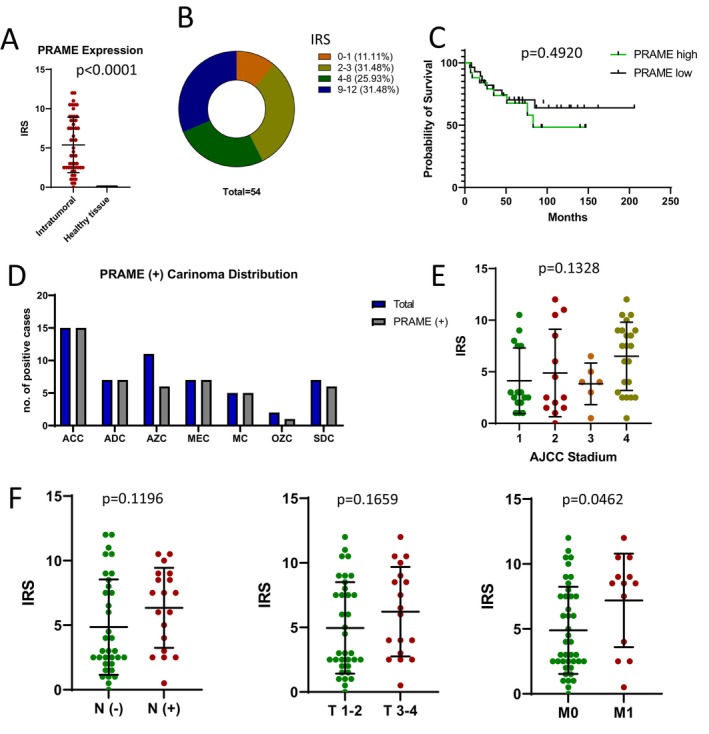
(A) PRAME expression in SGC vs. healthy salivary gland tissue. (B) Distribution of PRAME expression across 54 samples. (C) Kaplan–Meier overall survival analysis comparing PRAME‐high (IRS > 5.4) and PRAME‐low (IRS < 5.4) SGC groups. (D) PRAME positive carcinoma distribution across different histological subtypes: ACC: Adenoid Cystic Carcinoma, AZC: Acinic Cell Carcinoma, ADC: Adenocarcinoma, NOS, MEC: Mucoepidermoid Carcinoma, MC: Myoepithelial Carcinoma, OZC: Oncocytic Carcinoma, SDC: Salivary Duct Carcinoma. (E) Correlation of PRAME IRS with AJCC staging. (F) Correlation of PRAME IRS with *N*− vs. *N*+ status, T1‐2 vs. T3‐4, and M0 vs. M1 status. The respective *p*‐values are indicated within the figures.

PRAME expression in SGC cells was significantly higher compared to normal salivary gland tissue, with nearly 90% of tumor cells showing positive PRAME staining (*p* < 0.0001). Specifically, 31.5% of cases exhibited an IRS of 2–3, 25.9% had an IRS of 4–8, and 31.5% demonstrated strong expression with an IRS of 9–12 (Figure 3B). Immunohistochemical analysis revealed that PRAME expression in SGCs was predominantly nuclear, with variable cytoplasmic staining depending on the histological subtype. Kaplan–Meier survival analysis revealed no statistically significant difference in overall survival (OS). A trend toward better OS in the PRAME‐low group was observed, with survival rates exceeding 70% compared to 50% in the PRAME‐high group after 100 months of follow‐up. PRAME expression was detected in tumors across all histological subtypes (Figure 3D).

Analysis of PRAME expression in relation to AJCC stages using ordinary one‐way ANOVA did not reach statistical significance (*p* = 0.1328), but a trend indicated increased PRAME expression in higher tumor stages (Figure 3E). Further subgroup analyses using the Mann–Whitney *U* test revealed that PRAME expression tended to be higher in node‐positive cases (IRS 6.3) compared to node‐negative cases (IRS 4.8; *p* = 0.1196). Similarly, larger tumors (T3–4) tended to show higher PRAME expression (IRS 6.2) compared to smaller tumors (T1–2, IRS 5.0, *p* = 0.1659). A statistically significant difference in PRAME expression was observed between non‐metastatic (M0, IRS 4.9) and metastatic (M1, IRS 7.2) cases (*p* = 0.0462), highlighting a potential association between PRAME expression and metastatic disease (Figure 3F).

### 3.3 FOLR1 And CLDN18.2 Are Overexpressed in SGC but Not Associated With Disease Progression

FOLR1 and CLDN18.2 were both significantly overexpressed in SGC tissue compared to normal salivary gland tissue, with FOLR1 showing a more pronounced difference (mean IRS: 1.26; *p* < 0.0001 vs. 0.25 for CLDN18.2; *p* = 0.0001). FOLR1 was positive in 40% of cases, while CLDN18.2 was positive in only 6% of cases, indicating a lower prevalence of CLDN18.2 expression in salivary gland carcinomas. Notably, IRS < 1 was considered negative for all biomarkers, making most of the CLDN18.2 stainings negative.

**FIGURE 4:**
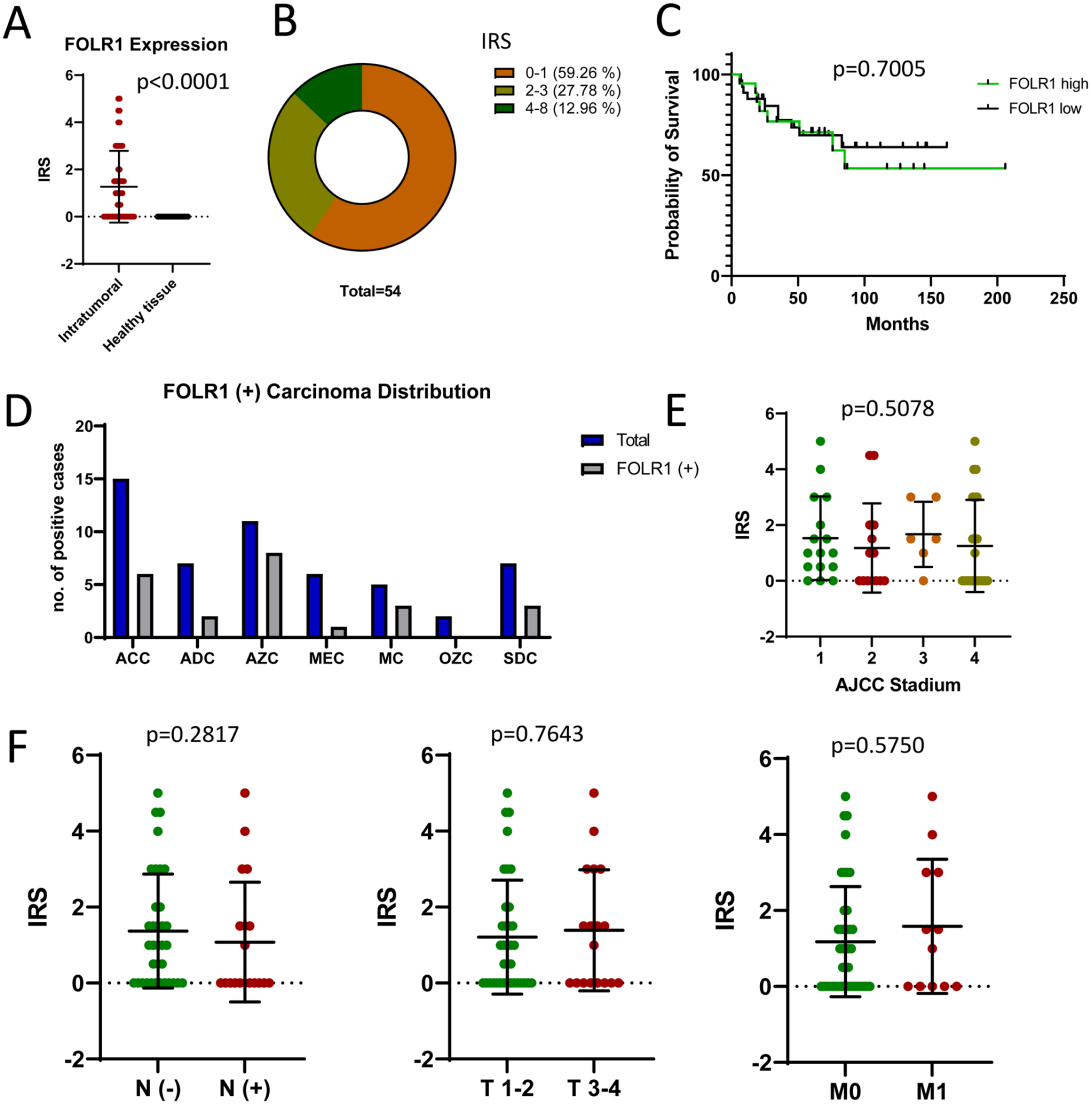
(A) FOLR1 expression in SGC vs. healthy salivary gland tissue. (B) Distribution of FOLR1 expression across 54 samples. (C) Kaplan–Meier overall survival analysis comparing FOLR1‐high (IRS > 1.3) and FOLR1‐low (IRS < 1.3) groups. (D) FOLR1‐positive carcinoma distribution across different histological subtypes: ACC: Adenoid Cystic Carcinoma, AZC: Acinic Cell Carcinoma, ADC: Adenocarcinoma, NOS, MEC: Mucoepidermoid Carcinoma, MC: Myoepithelial Carcinoma, OZC: Oncocytic Carcinoma, SDC: Salivary Duct Carcinoma. (E) Correlation of FOLR1 IRS with AJCC staging. (F) Correlation of FOLR1 IRS expression with *N*− vs. *N*+ status, T1‐2 vs. T3‐4, and M0 vs. M1 status. The respective *p*‐values are indicated within the figures.

**FIGURE 5:**
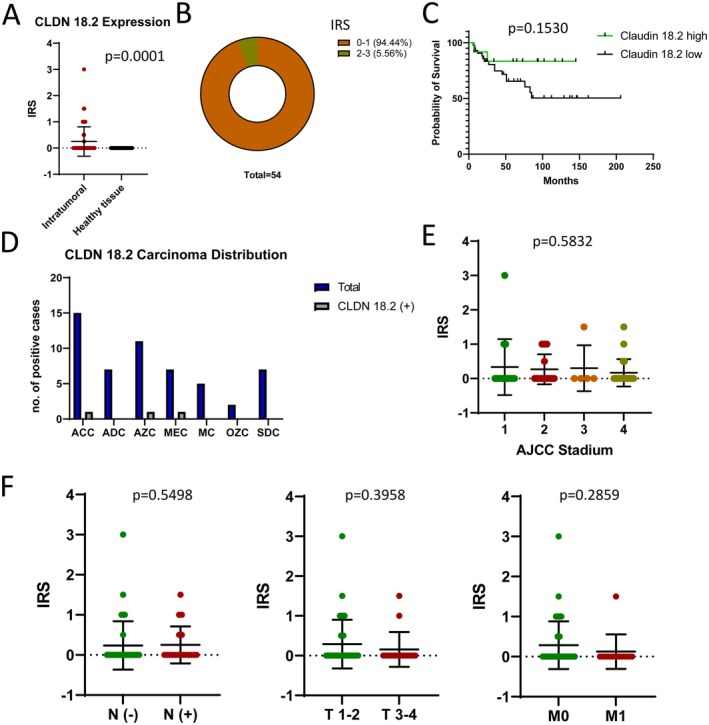
(A) CLDN18.2 expression in SGC vs. healthy salivary gland tissue. (B) Distribution of CLDN18.2 expression across 54 samples. (C) Kaplan–Meier overall survival analysis comparing CLDN18.2‐high (IRS > 0.3) and CLDN18.2‐low (IRS < 0.3) groups. (D) CLDN18.2‐positive carcinoma distribution across different histological subtypes: ACC: Adenoid Cystic Carcinoma, AZC: Acinic Cell Carcinoma, ADC: Adenocarcinoma, NOS, MEC: Mucoepidermoid Carcinoma, MC: Myoepithelial Carcinoma, OZC: Oncocytic Carcinoma, SDC: Salivary Duct Carcinoma. (E) Correlation of CLDN18.2 IRS with AJCC staging. (F) Correlation of CLDN18.2 IRS expression with N− vs. *N*+ status, T1‐2 vs. T3‐4, and M0 vs. M1 status. The respective *p*‐values are indicated within the figures.

Kaplan–Meier survival analysis revealed no statistically significant difference in overall survival between FOLR1‐high (IRS > 1.3) and FOLR1‐low (IRS < 1.3) groups (*p* = 0.7005) or between CLDN18.2‐high (IRS > 0.3) and CLDN18.2‐low (IRS < 0.3) groups (*p* = 0.1530). Similarly, neither biomarker showed a significant association with positive lymph node status or advanced disease stages, as shown in Figures 4 and 5.

Analysis of FOLR1 and CLDN18.2 expression in relation to AJCC staging using one‐way ANOVA did not reveal significant differences (*p* = 0.1328 for FOLR1, *p* = 0.5078 for CLDN18.2). Subgroup analyses using the Mann–Whitney *U* test also showed no significant correlations with nodal status (N− vs. N+), tumor size (T1‐2 vs. T3‐4), or metastatic status (M0 vs. M1) for either biomarker.

### 3.4 Entity‐Specific Analysis Revealed Highest PRAME Expression in ACC and MEC

Entity‐specific analysis revealed marked differences in PRAME expression across SGC subtypes. High PRAME expression (IRS 9–12) was most frequent in adenoid cystic carcinoma (ACC; 47%) and mucoepidermoid carcinoma (MEC; 43%), while moderate expression (IRS 4–8) was also commonly observed in these entities, as demonstrated in Table 2. In contrast, FOLR1 expression was predominantly low (IRS 0–1) across all subtypes, and CLDN18.2 expression was largely absent, with most cases showing IRS 0–1 regardless of histology. These findings underscore PRAME as the most consistently expressed target among the three markers evaluated, particularly in ACC and MEC.

**TABLE 2:** Distribution of PRAME, FOLR1, and CLDN18.2 expression across SGCs subtypes assessed by IRS. Expression levels are categorized as negative (IRS 0–1), low (IRS 2–3), moderate (IRS 4–8), and high (IRS 9–12).

	IRS	PRAME	FOLR1	CLDN18.2
ACC (*n* = 15)	0–1	0 (0%)	10 (67%)	14 (93%)
	2–3	4 (27%)	3 (20%)	1 (7%)
	4–8	4 (27%)	3 (20%)	0 (0%)
	9–12	7 (47%)	0 (0%)	0 (0%)
ADC, NOS (*n* = 8)	0–1	0 (0%)	6 (75%)	8 (100%)
	2–3	3 (38%)	2 (25%)	0 (0%)
	4–8	4 (50%)	0 (0%)	0 (0%)
	9–12	1 (13%)	0 (0%)	0 (0%)
AZC (*n* = 11)	0–1	4 (36%)	6 (55%)	10 (91%)
	2–3	6 (55%)	2 (18%)	1 (9%)
	4–8	0 (0%)	3 (27%)	0 (0%)
	9–12	1 (9%)	0 (0%)	0 (0%)
MEC (*n* = 7)	0–1	0 (0%)	7 (100%)	7 (100%)
	2–3	1 (14%)	0 (0%)	0 (0%)
	4–8	3 (43%)	0 (0%)	0 (0%)
	9–12	3 (43%)	0 (0%)	0 (0%)
MC (*n* = 5)	0–1	0 (0%)	5 (100%)	5 (100%)
	2–3	2 (40%)	0 (0%)	0 (0%)
	4–8	3 (60%)	0 (%)	0 (0%)
	9–12	0 (0%)	0 (%)	0 (0%)
SDC (*n* = 7)	0–1	1 (14%)	4 (57%)	7 (100%)
	2–3	1 (14%)	3 (43%)	0 (0%)
	4–8	5 (71%)	0 (0%)	0 (0%)
	9–12	0 (0%)	0 (0%)	0 (0%)
OZC (*n* = 2)	0–1	1 (50%)	2 (100%)	2 (100%)
	2–3	0 (0%)	0 (0%)	0 (0%)
	4–8	0 (0%)	0 (0%)	0 (0%)
	9–12	1 (50%)	0 (0%)	0 (0%)

*Note:* Data are shown as the number of cases with corresponding percentages within each histological subtype. Colours indicate increasing IRS scores, ranging from light brown (IRS 0–1) to intense brown (IRS 9–12).

## 4 Discussion

SGCs represent a challenging and recurrent malignancy with limited therapeutic options. Emerging immunotherapeutic strategies have thus far demonstrated only modest efficacy. To identify potential molecular targets for future therapies, this study investigated the expression patterns and clinical significance of PRAME, FOLR1, and CLDN18.2 in SGCs. The results underscore PRAME as a promising tumor‐associated biomarker, with widespread overexpression in SGCs (89% positivity) and a significant association with metastatic disease (*p* = 0.0462). While no survival benefit was observed for PRAME‐low groups, a trend toward improved overall survival in these cases needs further exploration in larger patient cohorts. In contrast, FOLR1 and CLDN18.2 showed low expression levels in our SGC cohort, with CLDN18.2 positivity in only 3/54 tumors (6%), making them less suitable for diagnostic purposes and therapeutic strategies.

The present study especially highlights the potential of PRAME as a therapeutic target in SGCs. Its high expression across all histological subtypes and association with advanced disease stages suggest that it could serve as a promising target for immunotherapeutic strategies. For instance, T‐cell‐based therapies, such as PRAME‐specific chimeric antigen receptor (CAR) T cells or peptide vaccines, have shown efficacy in other PRAME‐expressing malignancies, including melanoma and acute myeloid leukemia [28] as shown by Kirkey et al. and Blount et al. [29, 30]. While PRAME‐targeted vaccines and CAR T‐cells face challenges (e.g., limited CD8+ responses and intracellular antigen localization), TCR‐engineered T‐cells and ImmTACs (Immune mobilizing monoclonal T‐cell receptors Against Cancer) like brenetafusp (IMC‐F106C) show promising results, with phase I trials demonstrating 58% disease control in advanced melanoma. Ongoing phase III trials (NCT02743611, PRISM‐MEL‐301) aim to validate PRAME‐directed therapies in combination with checkpoint inhibitors for improved solid tumor outcomes. The high prevalence of PRAME expression in SGCs, particularly in aggressive subtypes like adenoid cystic carcinoma and advanced tumor stages, supports its suitability for targeted interventions; however, clinical trials are needed to validate this approach in SGCs.

Although not further analyzed due to limited sample size, PRAME immunohistochemistry notably highlighted areas of perineural invasion in ACC. Given the established prognostic impact of perineural invasion, this finding may support the potential diagnostic relevance of PRAME IHC and warrants further investigation in larger, stratified cohorts.

Despite the fact that FOLR1 was expressed at significantly higher levels in SGC tissue compared to healthy salivary gland tissue, its clinical significance remains questionable. FOLR1 positivity in 40% of our cases suggests a potential niche role in targeted therapies, such as folate receptor‐alpha (FRα)‐directed antibody‐drug conjugates (e.g., mirvetuximab soravtansine), which are under investigation in ovarian and endometrial cancers [31]. Notably, FOLR1 expression in SGCs was predominantly cytoplasmic, with only limited membranous localization, suggesting restricted accessibility for antibody–drug conjugate–based therapies that depend on membrane‐bound target antigens. However, the lack of correlation between FOLR1 expression and disease progression or survival in SGCs tempers enthusiasm for its utility. Conversely, the extremely low prevalence of CLDN18.2 positivity (3/54 tumors) precludes meaningful clinical interpretation, despite its role in gastrointestinal cancer therapeutics [32, 33].

The study's retrospective design and limited sample size (*n* = 54) restrict the statistical power, particularly for rare subtypes and biomarkers like CLDN18.2. The low event rate (e.g., only 3 CLDN18.2‐positive cases) further reduces the generalizability of findings. Additionally, the use of immunohistochemistry (IHC) introduces potential variability in scoring, though standardized IRS criteria mitigated this risk. Long‐term follow‐up data and multi‐institutional validation are needed to confirm the prognostic trends observed for PRAME. Due to the rarity and high heterogeneity of salivary gland malignancies, multi‐center trials would be necessary to validate our findings and create a basis for initial clinical trials.

Furthermore, one limitation of this study is the histological heterogeneity of salivary gland carcinomas and the relatively small cohort size, which limits the power of subtype‐specific analyses. Future studies with larger, histologically stratified cohorts are needed to validate and expand upon these results. In the present study, statistical analyses were therefore performed on the pooled cohort, and this limitation should be considered when interpreting associations between target expression and histopathological features. Only primary tumors were assessed in this study, and evaluation of metastatic samples will be important in future studies to better guide targeted therapies.

Based on our findings, future research should prioritize PRAME as a potential therapeutic target in preclinical models of SGCs, as, e.g., reported by Ikeda et al. [34], given its near‐universal expression in SGC and association with distant metastasis. Importantly, such investigations should increasingly incorporate advanced three‐dimensional (3D) salivary gland cancer models, which more accurately recapitulate tumor architecture, immune evasion, and tumor–microenvironment interactions than conventional two‐dimensional cultures. Recent studies have demonstrated the value of 3D models in salivary gland carcinomas, including adenoid cystic carcinoma, by revealing mechanisms of immune invisibility and suppression of anti‐tumor immune reactivity within a physiologically relevant microenvironment [35, 36]. For FOLR1, studies exploring its role in FRα‐targeted therapies—despite the lack of prognostic significance—could identify patient subgroups amenable to precision treatment. Conversely, CLDN18.2's minimal to no expression in SGCs suggests it is unlikely to be a priority for further investigation in this tumor type. Larger, prospective cohorts with molecular profiling (e.g., RNA sequencing) are critical to unraveling the heterogeneity of SGCs and identifying robust biomarkers.

## 5 Conclusion

Our study demonstrates that PRAME is highly expressed in the majority of salivary gland carcinomas, making it a promising candidate for targeted therapy approaches. In contrast, FOLR1 and CLDN18.2 demonstrate limited clinical relevance, though FOLR1 may retain niche therapeutic value. These findings underscore the need for biomarker‐driven multi‐center trials in SGCs, a rare and heterogeneous malignancy with highly limited treatment options.

## Author Contributions

**Lukas A. Brust:** conceptualization (equal), data curation (equal), formal analysis (equal), investigation (equal), methodology (equal), validation (equal), visualization (equal), writing – original draft (equal). **Jan Philipp Kühn:** writing – review and editing (equal). **Sandrina Körner:** writing – review and editing (equal). **Moritz Knebel:** writing – review and editing (equal). **Felix L. Braun:** writing – review and editing (equal). **Philippe Zeidan:** writing – review and editing (equal). **Silke Wemmert:** writing – review and editing (equal). **Bernhard Schick:** writing – review and editing (equal). **Sigrun Smola:** writing – review and editing (equal). **Mathias Wagner:** investigation (equal), methodology (equal), writing – review and editing (equal). **Martin Ertz:** methodology (equal), resources (equal), software (equal), writing – review and editing (equal). **Maximilian Linxweiler:** conceptualization (equal), data curation (equal), investigation (equal), methodology (equal), resources (equal), supervision (equal), validation (equal).

## Ethics Statement

The study was approved by the Saarland Ethics Review Board (index number 280–10) and conducted in accordance with the Declaration of Helsinki and relevant guidelines.

## Consent

All patients provided written informed consent for the scientific use of their tissue samples and clinical data.

## Conflicts of Interest

The authors declare no conflicts of interest.

## Data Availability

The data that support the findings of this study are available on request from the corresponding author. The data are not publicly available due to privacy or ethical restrictions.
